# The Role of a Navigational Radiofrequency Ablation Device and Concurrent Vertebral Augmentation for Treatment of Difficult-to-Reach Spinal Metastases

**DOI:** 10.3390/curroncol28050340

**Published:** 2021-10-08

**Authors:** Claudio Pusceddu, Davide De Francesco, Luca Melis, Nicola Ballicu, Alessandro Fancellu

**Affiliations:** 1 Regional Referral Center for Oncologic Disease, Department of Oncological and Interventional Radiology, Businco Hospital, A.O. Brotzu, 09100 Cagliari, Italy; clapusceddu@gmail.com (C.P.); Nicola.ballicu@aob.it (N.B.); 2 Institute for Global Health, UCL, Royal Free Campus Rowland Hill Street, London NW3 2PF, UK; d.defrancesco@ucl.ac.uk; 3 Division of Nuclear Medicine, Businco Hospital, Regional Referral Center for Oncologic Disease, A.O. Brotzu, 09100 Cagliari, Italy; doclucamelis@tiscali.it; 4 Unit of General Surgery, Department of Clinical and Experimental Medicine, University of Sassari, 07100 Sassari, Italy

**Keywords:** radiofrequency ablation, pain management, osseous metastasis, interventional oncology, vertebral augmentation

## Abstract

Aims: The purpose of this study was to assess the effectiveness of a navigational radiofrequency ablation device with concurrent vertebral augmentation in the treatment of posterior vertebral body metastatic lesions, which are technically difficult to access. Primary outcomes of the study were evaluation of pain palliation and radiologic assessment of local tumor control. Materials and Methods: Thirty-five patients with 41 vertebral spinal metastases involving the posterior vertebral body underwent computed tomography-guided percutaneous targeted radiofrequency ablation, with a navigational radiofrequency ablation device, associated with vertebral augmentation. Twenty-one patients (60%) had 1 or 2 metastatic lesions (Group A) and fourteen (40%) patients had multiple (>2) vertebral lesions (Group B). Changes in pain severity were evaluated by visual analog scale (VAS). Metastatic lesions were evaluated in terms of radiological local control. Results: The procedure was technically successful in all the treated vertebrae. Among the symptomatic patients, the mean VAS score dropped from 5.7 (95% CI 4.9–6.5) before tRFA and to 0.9 (95% CI 0.4–1.3) after tRFA (*p* < 0.001). The mean decrease in VAS score between baseline and one week follow up was 4.8 (95% CI 4.2–5.4). VAS decrease over time between one week and one year following radiofrequency ablation was similar, suggesting that pain relief was immediate and durable. Neither patients with 1–2 vertebral metastases, nor those with multiple lesions, showed radiological signs of local progression or recurrence of the tumor in the index vertebrae during a median follow up of 19 months (4–46 months) and 10 months (4–37 months), respectively. Conclusion: Treatment of spinal metastases with a navigational radiofrequency ablation device and vertebral augmentation can be used to obtain local tumor control with immediate and durable pain relief, providing effective treatment in the multimodality management of difficult-to-reach spinal metastases.

## 1. Introduction

**Figure 1:**
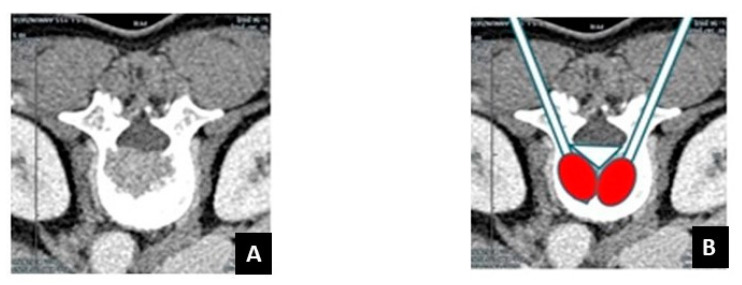
Limits of the use of conventional radiofrequency straight needles for the treatment of difficult-to-reach vertebral metastases. (**A**) Breast cancer metastasis in a 61-year-old patient. Axial CT scan shows a lytic lesion of in the posterior wall of the vertebral body L2 with cortical disruption. (**B**) Conventional RFA with straight needles is not able to ablate the entire lesion (white triangle).

**Figure 2:**
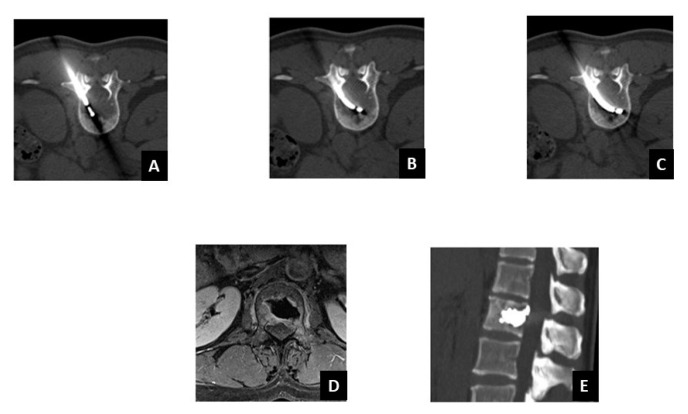
Advantages of the use of the navigational radiofrequency ablation device using steerable needles instead of conventional straight ones. L2 breast cancer metastasis (same patient from Figure 1). (**A**–**C**) Placement of targeted radiofrequency ablation device to obtain complete necrosis of the tumor lesion located in a difficult-to-access location. (**D**,**E**) Follow-up imaging after one month after the tRFA-VA treatment. (**D**) MRI showed a layer of granulation tissue around the ablated zone. This is the typical inflammatory reaction after tRFA ablation. (**E**) CT scan showed the right position of the polymethylmethacrylate used for VA. After tRFA, vertebroplasty was performed to strengthen the bone in order to avoid pathological fracture.

The incidence of bone metastases in patients with cancer is extremely high: 84% for prostate, 72% for breast, 60% for thyroid, 37% for kidney, 33% for pancreas, and 31% for lung [1,2]. In particular, vertebral metastases occur in approximately 60–70% of patients with a primary tumor [3]. This high frequency of spine involvement can be due to the high hematopoietic activity and vascularization of the vertebrae [4]. Metastatic spinal lesions can involve one or two vertebrae or, in some cases, can affect multiple vertebrae. Conventional treatment regimens such as radiotherapy, chemotherapy, and analgesic drugs may have shortcomings in the treatment of these lesions [4,5,6]. Surgical decompression and stabilization represent an option for patients with spine instability and neurologic symptoms, especially in those with a longer life expectancy [4,7]. Several tumor ablation techniques, such as cryoablation, radiofrequency, microwave ablation, and laser interstitial thermal therapy, have been developed for percutaneous treatment of vertebral metastases and osseus metastatic lesions in general, and each of these methods has specific indications and advantages [4,8,9,10,11]. In particular, radiofrequency ablation (RFA) associated to vertebral augmentation (VA) has been gaining popularity in the multidisciplinary treatment of spinal metastases without spinal cord compression [5,12,13,14,15]. In addition, the combined treatment with RFA and VA of spinal metastases improves oncological outcomes in comparison with vertebroplasty or radiofrequency ablation alone [2,4,5,11]. However, the standard RFA involves the use of straight needles through a transpeduncolar approach, which can be inadequate for obtaining ablation of tumor tissue located in difficult-to-reach metastases, such as those located in the posterior part of the vertebral body (Figure 1). Recently, this important disadvantage of the use of straight needles has been overcome by targeted radiofrequency ablation (tRFA). In tRFA, a navigational radiofrequency ablation device is used, which contains an articular extensible electrode that allows treatment of posterior spinal metastases with a curved needle (Figure 2) [4,5,13,14,15]. To date, few authors have reported on the use of a navigational radiofrequency ablation device for the treatment of difficult-to-reach spinal metastases, and many aspects need to be clarified [4,5,13,14,15] (Table 1). The purpose of this study was to assess the effectiveness of tRFA and VA using a navigational radiofrequency ablation device in the treatment of posterior vertebral body metastatic lesions that are technically difficult to access via a transpedicular approach. Primary outcomes of the study were evaluation of pain palliation, and radiologic assessment of local tumor control.

## 2. Material and Methods

### 2.1. Study Population and Inclusion Criteria

Patients who underwent minimally invasive CT fluoroscopy-guided percutaneous tRFA and VA between November 2013 and March 2017 were identified. Patients were included in the study if they had vertebral lesions from T1 to L5, with evidence of osteolytic or mixed metastatic lesions in the posterior vertebral body (i.e., in the posterior half of the vertebra), irrespective of whether pathologic fractures occurred. Patients with disruption of the posterior cortical wall were also included. Patients with metastases located in the spinal cord and/or nerve root compression were excluded.

### 2.2. Group Definition and Preoperative Assessment

Patients were classified into two groups according to the number of metastases located in the spine:

(1) Group A: patients with 1 or 2 vertebral lesions.
(2) Group B: patients having more than 2 metastatic spine lesions.

All patients were assessed prior to surgery with a CT scan or MRI and, in some cases, PET scan in order to study the metastatic vertebral lesion and to plan the percutaneous intervention. CT scan, MRI, or PET scan were performed at 1, 6, and 12 months post tRFA treatment to assess the local activity of the vertebral metastases and/or residual viable tumor, and/or recurrence/growth of the spinal metastases (Figure 2). Pain severity was documented using a visual analog scale (VAS—scaled from a minimum of 0 to a maximum of 10) [16] the day before the treatment, and then one week, six months, and twelve months after tRFA and VA treatment.

### 2.3. Target Radiofrequency Ablation (tRFA) and Vertebral Augmentation (VA) Procedure

**Figure 3:**
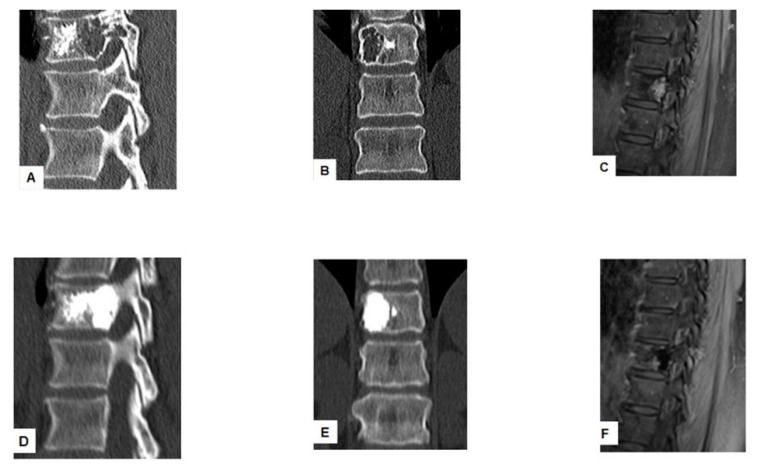
Successful treatment with tRFA and vertebroplasty with VA of a painful vertebral metastasis in a 74-year-old man with metastatic colon cancer. The lesion located in the posterior vertebral wall of T10 was initially treated with vertebroplasty (**A**,**B**). MRI scan T1 fat suppression shows progression of the lesion after vertebroplasty (**C**). tRFA was performed in the posterior vertebral body: axial and coronal CT scan reconstruction after tRFA followed by vertebroplasty (**D**,**E**). Postprocedural MRI scan T1 fat suppression after tRFA and vertebroplasty showed lack of contrast enhancement of the lesion (**F**).

Using CT fluoroscopy guidance (SOMATOM Sensation, Siemens, AG, Forchheim, Germany), the spine lesion was identified, measured, and marked to establish and validate the desired needle pathway. The procedures were performed according to the following steps: (i) anesthesia: conscious sedation with intravenous infusion of fentanyl citrate (0.1 mg/2 mL diluted with saline solution) followed by subcutaneous injection of lidocaine hydrochloride (2%) performed until the pedicle periosteal and the bone cortex. (ii) tRFA: under CT scan, a 10-G vertebroplasty needle was introduced and, coaxially to it, the bipolar tRFA electrode (STAR, Merit Medical Systems, Inc., South Jordan, UT, USA) was placed into the vertebral lesion to deliver the RF energy and ablate the metastases properly. The instrument has two thermocouples (the distal at 10 mm and the proximal at 15 mm from the ablation core) which actively monitor the temperature of the tumor avoiding undesired burns around the lesion. (iii) Vertebral augmentation: using the same vertebroplasty needle, in order to minimize cement leakage risk, ultra-high viscosity bone cement was inserted through a controller under low-pressure injection (STABILIT, Merit Medical Systems, Inc.) for optimal filling of the vertebral lesion (Figure 3).

### 2.4. Postoperative Follow Up

CT scan, MRI, or PET (in selected cases) were performed at 1, 6, and 12 months after tRFA-VA treatment to assess local activity of the vertebral metastases and/or residual viable tumor, as well as recurrence/growth of the spinal metastases. Tumor progression was defined as an increase of the size of metastasis on CT or MRI. Stable disease was considered as a stable metastasis diameter along with no contrast enhancement. Recurrence at follow-up imaging was defined as contrast enhancement or enlargement at the site of successfully treated lesion, and/or the occurrence of osteolytic lesions near the treated areas.

### 2.5. Statistical Analysis

Changes over time in the VAS score were evaluated using a linear mixed-effect model to account for the correlation between VAS scores within the same patient. Changes in the VAS score before and one week after tRFA were modeled with a dummy variable, while scores from one week after tRFA onwards were modeled as a linear function of time. All statistical analyses were performed using R Statistical Software (version 3.3.3) and *p* < 0.05 was considered significant. 

## 3. Results

**Figure 4:**
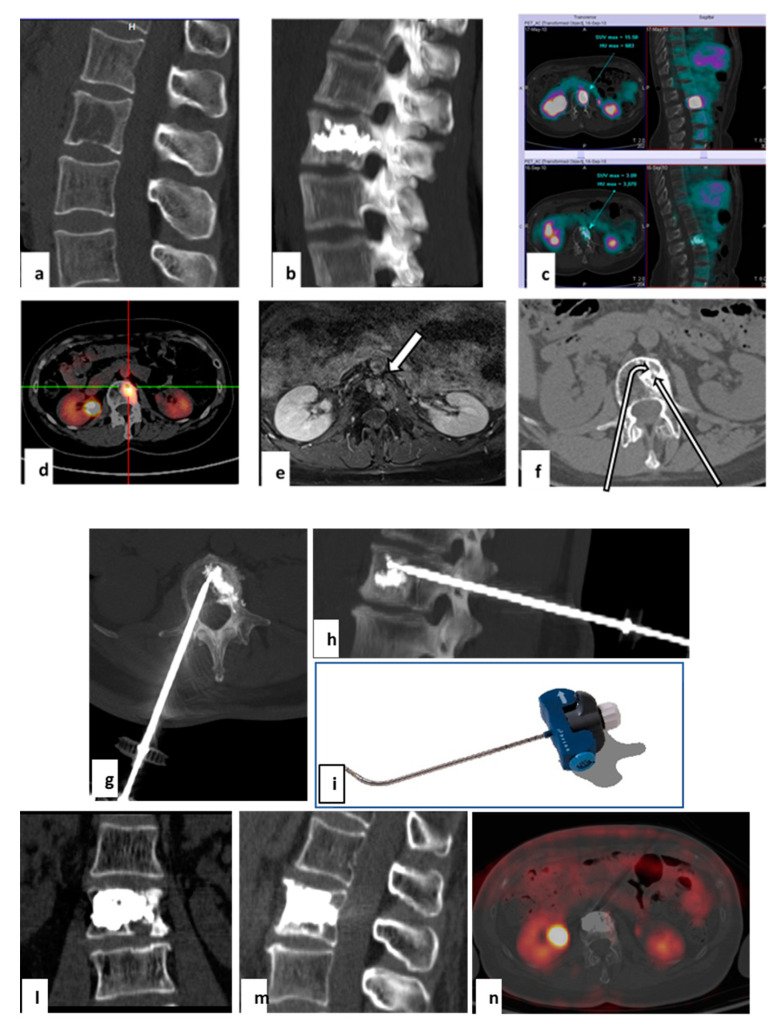
Treatment with tRFA and VA of a recurrent metastasis in a 57-year-old woman with a single L2 lytic metastasis from breast cancer. The metastasis had been treated previously with microwave ablation. Sagittal CT reconstruction showed a large lytic metastasis of L2 treated with microwave ablation (**a**). Sagittal CT obtained with MIP technique demonstrated the results of vertebral augmentation (**b**). PET-FDG performed before and 4 months after treatment demonstrates complete absence of metastasis uptake (**c**). Vertebral tumor recurrence 12 months after the microwave ablation treatment. FDG-PET demonstrated intense uptake of the vertebral body, anterior to the cemented area (**d**). T1 MRI with fat suppression acquired after gadolinium infusion revealed an area of contrast enhancement related to recurrence (**e**). Cement interposition hindered access to metastasis by left transpeduncular route with straight needle (straight arrow). The lesion can be reached by contralateral transpeduncular approach with a steerable needle (tSTAR) (**f**). Axial and coronal CT scans reconstructed with MIP technique (**g**,**h**) after tRFA navigation system placement (**i**). Coronal and sagittal CT scans after treatment of the second vertebroplasty (**l**,**m**). PET-FDG performed after 6 months demonstrates absence of metastasis uptake (**n**).

**Figure 5:**
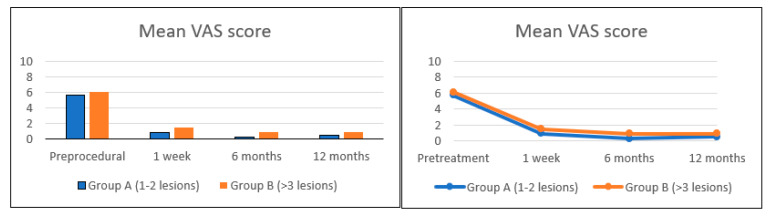
Evaluation of VAS score in symptomatic patients across Group A and Group B, before tRFA, 1 week, 6 months, and 12 months after tRFA.

Forty-one vertebral metastases were treated in thirty-five patients (22 females and 13 males, median (IQR) age 59 (56–70) years). Breast cancer was the most common type of primary tumor (*n* = 16, 46%), followed by lung (*n* = 5, 14%), colon (*n* = 4, 11%), prostate (*n* = 3, 9%), and kidney (*n* = 2, 6%). None of the patients had signs of neurological deficits, nor required an open surgical stabilization. Twenty-one (60%) patients belonged to Group A (1 or 2 vertebral metastatic lesions) (Table 2), the remaining fourteen (40%) to Group B (multiple metastases in the spine) (Table 3). In one case, tRFA and VA were successfully used after a tumor recurrence after a vertebral microwave ablation (Figure 4). Two patients belonging to Group A were asymptomatic. All the procedures were uneventful and well tolerated, without complications related to tRFA and concurrent VA. Asymptomatic cement leakage occurred in 3 of 41 treated vertebrae (7.3%). Ten patients (29%) received radiotherapy before tRFA, five (14%) after tRFA, and twenty (57%) patients did not receive radiotherapy at all. No local relapse nor tumor progression was observed in both Group A during a median follow up of 12 months (4–46 months), and Group B during a median follow up of 10.5 months (4–37 months). Eight patients in Group B and two patients in Group A died as a result of disease progression. In the 33 patients with painful metastases, the mean value of VAS score before the procedure, after 1 week, 6 months, and 12 months was 5.7 ± 1.6, 0.9 ± 0.7, 0.3 ± 0.4, 0.5 ± 0.6, and 6.1 ± 1.8, 1.5 ± 1.0, 0.9 ± 0.7, 0.9 ± 0.7 in Group A and Group B, respectively (Figure 5). Considering the patients as a whole, the mean VAS score dropped from 5.7 (95% CI 4.9–6.5) before tRFA to 0.9 (95% CI 0.4–1.3) after tRFA (Figure 5). The mean decrease in the VAS score between baseline and follow up was 4.8 (4.2–5.4, *p* < 0.0001). With the numbers available, we found no difference in the VAS score over time from one week up to one year after tRFA and VA, suggesting that pain relief was immediate and durable. There was no difference in the VAS scores before and after surgery between the two groups of the study. Use of steroids and non-steroidal anti-inflammatory drugs taken by patients in both groups were reduced or completely suspended after the procedure. Patient characteristics and results are summarized in Table 2 and Table 3.

## 4. Discussion

The use of tRFA associated to vertebroplasy with VA has been gaining importance in the management of spine metastases [5,13,14,17]. However, classical RFA with straight needles usually permits the treatment of lesions located in the anterior part of the vertebral body. In this study, we have evaluated the usefulness of tRFA with a navigation radiofrequency ablation device, which overcomes some limitations of conventional RFA [5,13,14,15]. Our results demonstrated that tRFA with a dedicated navigation radiofrequency device, with concurrent VA, can obtain satisfactory results in terms of pain reduction and local tumor control.

This study has some limitations, the main being its retrospective nature and the small sample size. Moreover, the overlapping with radiation therapy in some patients and the use of drugs for pain relief, may have influenced the VAS score. However, this study has some points of strengths, in that it supports the use of a navigation radiofrequency ablation device for the treatment of metastases located in the posterior part of the vertebrae. As we reported in Table 1, only few studies have addressed the use of that approach, and some authors limited their experience to patients with vertebral osteoid osteoma [15]. To the best of our knowledge, this is the first single-institution report from a European institution on the use of a navigational system for the tRFA of vertebral metastases.

Spinal metastases represent a complex clinical scenario in patients affected from cancer, especially in those with decreased life expectancy [5,14,18]. Those lesions can result in severe complications such as pain, vertebral fractures, hypercalcemia, spinal instability, and neurological symptoms due to spinal cord and/or nerve root compression [13,14,15,19]. tRFA with concurrent vertebroplasty has shown effectiveness for vertebral lesions without spinal cord compression. Normally, RFA treatment in the spine is used for lesions located in the anterior vertebral body and far from neurovascular structures, where a conventional RF ablation straight device can easily reach the metastases [13,20]. The navigational radiofrequency ablation device differs in that it has the ability to properly curve the needle, which allows navigation of the instrument; this permits the obtaining of necrosis of tumor cells even in difficult locations of the vertebral body. Furthermore, the steerable needle is able to approach vertebral lesions that are technically challenging to access with straight needles, such as metastases close to the posterior vertebral wall and spinal cord. To note, all patients in the present study had one or more metastatic lesions located in the posterior vertebral body, without signs of spinal cord compression. The latter commonly represents a contraindication for percutaneous ablation as upfront treatment, being corticosteroids, surgery, radiotherapy, and systemic therapy the preferred options [12,21,22].

One of the purposes of our work was to evaluate the role of tRFA with concurrent VA in the control of pain, using a dedicated navigational device. Cancer-induced bone pain recognizes multiple mechanisms, including disruption of bone homeostasis, release of neurochemicals that modulate pain, and microglia activation. Furthermore, instability of a vertebral body usually causes pain, which is poorly localized, and sometimes becomes intractable [23]. It has shown that VA is an effective option for pain control and stabilization of pathologic fractures of the spine [5,14,24,25,26]. Progressive pain is a frequent symptom with important repercussions in the quality of life. tRFA in combination with VA is the preferred approach in certain situations, such as single vertebral metastases, spinal active residual tumor tissue, and occurrence of vertebral compression fractures with cortical interruptions after radiotherapy [24,25,26]. However, our results confirm that pain control is achieved by using tRFA-VA also in patients with multiple localizations. In fact, the VAS scores indicated that pain symptoms were durably relieved in both the study groups. To note, the mean decrease in the VAS score between baseline and follow up was 4.8 (*p* < 0.0001), and no difference was observed in the VAS score over time from one week up to one year after tRFA and VA, suggesting that pain relief was immediate and durable. These findings were consistent to previous studies. In the multicentric study from Anchala and colleagues, significant (*p* < 0.01) decrease in the VAS scores at one week, one month, and six months was observed, although only 62% of the spinal lesions in the largest institution participating in the study was located in the posterior vertebral body [14]. Reyes et al. reported that mean VAS scores decreased from 7.9 pre-procedure to 3.5 post-procedure (*p* < 0.0001) in their multicentric study involving almost 50 patients [4].

The second purpose of the present study was to evaluate the metastatic lesions in terms of radiological local control after tRFA and VA treatment. Our results reinforce the concept that treating patients with a navigational radiofrequency device with the goal to achieve tumor control is feasible [5]. Twenty-one patients with 1 or 2 metastatic lesions showed no local relapse or tumor progression during a median follow up of 19 months. Similarly, none of the 14 patients with multiple lesions developed local relapse or tumor progression after a follow up of 10 months. These findings may be partially explained by the small sample size as well as the short follow-up duration. However, data regarding follow-up imaging and local relapse after the use of a navigational radiofrequency device for spinal metastases are very scarce. In the study from Anchala et al., only 13 out of 92 patients received follow-up postoperative imaging, of whom 3 developed tumor progression at the treated site [14]. Hillen et al. reported on the use of a navigational radiofrequency ablation device on 26 patients with metastatic posterior vertebral body. Of them, 13 patients underwent follow-up imaging, of whom 3 developed tumor progression at the treated site [13]. In the multicentric study from Reyes et al., 49 patients received navigational radiofrequency ablation and VA for vertebral metastases. Among those who had postoperative follow-up imaging, MRI showed tumor extension into epidural space and neural foramen in one case, and a new epidural extension from a different primary tumor in another case [4].

As described elsewhere, the goal of performing tRFA before vertebroplasty is to destroy tumor tissue, to reduce the intrametastatic pressure, and to thrombose intravertebral venous plexus [27]. The purpose of vertebroplasty with VA is to stabilize the vertebra by stabilization of vertebral microfractures with cement [28]. Literature reports described that polymethylmethacrylate alone and the exothermic reaction generated during its polymerization are not able to completely destroy tumor cells [8,29]. Some authors even reported that the injection of the cement in a metastatic vertebral body increases the intrametastatic pressure with a risk of dissemination of tumor cells [30]. Four patients out of thirty-five enrolled in this study were treated with tRFA after they underwent previous vertebroplasty alone, and they developed new signs or reappearance of the metastatic vertebral lesion associated with pain (Figure 3). Although this phenomenon involves only a few patients, it underlines that vertebroplasty alone is not efficient in local tumor control. Two (2) asymptomatic patients were treated with tRFA in combination with vertebroplasty, which is a further example of the effectiveness of the ablation treatment. In fact, a goal of tRFA-VA is to obtain local control of the bone disease by destroying the spinal metastases, avoiding extension into the neural structures, and limiting vertebral fracture risk associated with radiotherapy, as reported in the literature [24,25]. Furthermore, this approach may avoid skeletal-related events associated with standard treatments (chemotherapy, radiotherapy, bisphosphonates, and surgery), which include pain, risk of fracture, and nerve or spinal cord compression [27]. In addition, it has been reported that tRFA-VA may improve anti-tumor local response in association with radiotherapy [31].

External-beam radiation therapy is another option to treat pain and achieve local control and represents the standard treatment for pain relief and local control of spinal metastases [7,12,13]. However, radiotherapy has certain limitations as only a few tumor types are considered to be highly radiosensitive. Furthermore, pain relief obtained with radiotherapy may be transient and delayed [13,27,32,33]. In our study, 10 patients received low-dose palliative radiotherapy before tRFA and vertebroplasty treatment. Unfortunately, our data did not provide insight about the best timing of tRFA treatment in the oncologic patient care pathway. Optimal radiotherapy timing in combination of tRFA and vertebroplasty remains unclear and needs to be assessed in future studies. Interestingly, the effectiveness of radiation therapy after surgical fixation of impending or actual pathologic fractures in the long bones has been questioned. A metanalysis involving two studies has shown conflicting results, and the authors concluded that data were insufficient to conclude whether postoperative RT after surgical stabilization should be standard care for long bone metastases [34]. Nonetheless, in current literature there is evidence that both radiotherapy and percutaneous termoablation modalities such as tRFA have been shown to be safe and effective in the treatment of painful vertebral metastases, and thus a synergic effect can be hypothesized in a multidisciplinary approach to vertebral metastases [5,35]. Our data corroborate that the sequence tRFA-VA and radiotherapy can be helpful in cases where pain still persists after percutaneous treatment; in fact, in five cases of the present series where cancer-related pain was consistent, radiotherapy was used to optimize pain control after tRFA-VA.

It is our thought that the goal of the tRFA in treating patients with metastatic spinal tumors should be not only the pain relief, but also the achievement of an oncological outcome in terms of stopping lesions from spreading into the rest of the vertebral body to avoid neurological damage. Our study indicates that percutaneous treatment of spinal metastases with tRFA and vertebroplasty can be used in a multimodality therapeutic approach. In addition, it may bridge the gaps reported for conventional treatment methods in vertebral metastases management. Radiation therapy, chemotherapy, “open” spinal surgeries, vertebroplasty, and “conservative” treatment are treatments that must be tailored for each patient, considering the nature of the tumor, life expectancy, pain, metastases at high risk of fracture, and compression of neurovascular structures [36].

In conclusion, tRFA using a dedicated radiofrequency ablation device, with concurrent VA, can achieve satisfactory results in terms of pain palliation and local tumor control in patients with posterior vertebral body metastases. That approach may widen the field of application of termoablation in the management of spinal metastases. Further studies with larger cohorts are necessary to establish the optimal role of tRFA and vertebroplasty in association with radiotherapy in the multidisciplinary treatment of vertebral metastases.

## Figures and Tables

**Table 1:** Summary of studies reporting on the use of a navigational radiofrequency ablation device for the treatment of spinal lesions.

Author	Country	Year	No of Patients/No of Treated Lesions	Inclusion Criteria	Treatment of Spinal Lesions	Main Conclusion(s)
Anchala [14] *	US	2014	92/128	Spine metastatic lesions	tRFA ± VA	The STAR System is an RFA device that was safely and effectively used in the treatment of spine metastatic osseous lesions.
Hillen [13]	US	2014	26/47	Posterior vertebral body tumors	tRFA + VA	Targeted RFA with a newly developed articulating device is both feasible and safe for the treatment of painful posterior vertebral body metastatic tumors.
Reyes [4] *	US/Italy	2017	49/72	Vertebral metastases	tRFA + VA	tRFA followed by vertebral augmentation in malignant vertebral lesions resulted in significant pain reduction and functional status improvement.
Tomasian [15]	US	2018	7/7	Spinal osteoid osteomas	tRFA	Safe and effective percutaneous CT-guided radiofrequency ablation of spinal osteoid osteomas can be performed using a targeted navigational bipolar electrode system.

* Multicentric study.

**Table 2:** Group A (patients with one or two vertebral metastatic lesions).

3	Primary Tumor	Baseline Imaging	Site of Treated Vertebrae	Baseline LXD (cm × cm)	RT before/after tRFA	Complication	VAS before/after tRFA	VAS 6 Months	VAS 12 Months	Imaging Post tRFA	Status-Follow Up (Months)
49-F	Melanoma	CT-PET	T10	1.5 × 1.2	No	no	3/0	0	0	CT-Scintigraphy	alive—46
51-F	Breast	CT- PET	L1	3.2 × 2	No	no	3/0	0	0	MRI	alive—45
39-F	Breast	Scintigraphy-MRI	T8	1.8 × 1.6	After	no	4/0	0	0	MRI	alive—40
58-F	Breast	PET-CT-MRI	L2, L4	2.1 × 1.8–2 × 1.4	After	no	4/0	0	0	MRI	alive—40
47-F	Breast	MRI-CT-PECT	T9	2.6 × 2	Before	no	5/0	0	0	CT-Scintigraphy	alive—34
57-F	Breast	CT-MRI-PET	L2	2 × 1	Before	no	2/0	0	0	MRI-PET	alive—28
78-M	Prostate	CT	T3	1.8 × 1.5	No	no	5/0	0	0	CT-MRI	death—7
66-F	Breast	CT-MRI-PET	L3	1.2 × 1.3	No	no	5/0	0	0	MRI-PET	alive—22
72-F	Colon	CT-PET	L3	2.4 × 2	Before	no	6/0	0	0	CT	death—10
65-F	Breast	CT-PET	L3	2.3 × 2	Before	no	7/2	0	0	MRI	alive—21
70-M	Prostate	CT-PET	T10	2 × 2.2	No	no	6/0	0	0	CT-MRI	alive—19
70-M	Colon	CT	T12	2.9 × 2.4	Before	no	4/0	0	0	MRI	alive—19
56-F	Breast	CT	L3	3 × 2.3	No	no	7/2	0	0	CT-MRI	alive—19
62-F	Lung	CT-MRI	L1	2.4 × 1.6	No	no	7/0	0	0	CT	alive—15
76-F	Breast	CT	T7, T10	2.1 × 1.8–2 × 1	No	no	8/2	0	0	CT	alive—13
56-F	Breast	CT-MRI	T9	1.3 × 1.1	No	no	4/0	0	0	CT-PET	alive—12
57-F	Breast	CT-MRI-PET	L2	1 × 1	No	no	4/0	0	0	MRI-PET	alive—8
71-F	Multiple myeloma	CT-MRI	S1	5 × 4	No	no	7/2	0	N/A	CT-MRI	alive—10
54-M	Lung	CT	T8	3.2 × 1.2	No	no	8/0	N/A	N/A	CT	alive—4
57-F	Breast	CT	T6, T8	2 × 2–2 × 2.05	No	no	8/0	N/A	N/A	CT	alive—4
55-M	Prostate	CT	T11	3 × 3	No	no	6/2	N/A	N/A	CT	alive—4

**Table 3:** Group B (patients with more than two vertebral metastatic lesions).

Age-Gender	Primary Tumor	Baseline Imaging	Site of Treated Vertebrae	Baseline LXD (cm × cm)	RT before/after tRFA	Complication	VAS before/after tRFA	VAS 6 Months	VAS 12 Months	Imaging Post tRFA	Status-Follow Up (Months)
67-F	Breast	CT-PET-MRI	L2	1.9 × 2	before	No	4/0	0	0	PET	alive—33
57-F	Breast	CT-MRI	T8	1.06 × 1	No	No	5/0	0	0	MRI-CT	alive—37
59-M	Lung	CT	L1	3 × 3.5	before	No	6/0	0	0	CT-Scintigraphy	death—4
57-M	Colon	CT	L5	4.5 × 3.8	after	No	8/3	3	N/A	MRI	death—12
70-F	Thyroid	CT-Scintigraphy	T10	2.3 × 0.7	No	No	3/0	0	0	MRI-Scintigraphy	alive—24
68-M	Kidney	CT	T11, L2	2.4 × 2.2–2.4 × 1.5	before	posterior leakage	8/2	3	5	MRI	death—19
41 -F	Sarcoma	CT	L3, L4	2.2 × 1.7–2 × 1.4	No	No	8/3	2	0	CT	death—12
79-M	Lung	CT	T4	3.1 × 2.5	No	No	8/5	N/A	N/A	CT	death—4
40-M	Adrenal gland	CT	L1	2.9 × 3.1	No	No	7/1	0	0	CT	death—4
71-M	Lung	CT-PET	L4	4.6 × 3.7	After	posterior leakage	8.5/3	N/A	N/A	CT	death—5
66-M	Kidney	CT	T12	5 × 3	After	lateral leakage	9/3	0	0	CT	alive—11
65-M	Colon	CT-MRI	L1	1.8 × 1.3	before	No	6/0	0	0	CT	death—6
71-F	Breast	CT-MRI	L2, L3	3 × 2.7–2.9 × 2.8	before	No	6/0	3	3	CT	alive—10
58-F	Breast	CT	T12	1.7 × 1.5	No	No	6/0	N/A	N/A	CT	alive—5

## Data Availability

Data will be available under reasonable request.
